# Shared Decision-Making in Anesthesia in the Indian Context: A Cross-Sectional Study

**DOI:** 10.7759/cureus.96552

**Published:** 2025-11-11

**Authors:** Preethi Kuryan, Samuel Santhosh, Bernice Theodore, Meghna Jiwanmall, Aparna Williams

**Affiliations:** 1 Anesthesiology, Christian Medical College, Vellore, Vellore, IND; 2 Orthopedics, Christian Medical College, Vellore, Vellore, IND

**Keywords:** anesthesia technique, limb surgery, patient preference, patient role, shared decision-making

## Abstract

Background

Patient participation in the decision-making process is an important component of modern evidence-based medicine. In this study, we assessed the involvement of patients in making a decision while choosing an anesthesia technique for elective surgery of a single limb.

Methodology

This cross-sectional study at a tertiary care institute enrolled patients planned for elective surgery of a single limb. Questionnaires were employed to assess patients’ demographic details and the participants’ preferred technique of anesthesia for the surgery. On completion of pre-anesthesia evaluation, standardized questionnaires (Shared Decision-Making Questionnaire (SDM)-Q-9 for patients and SDM-Q-Doc for doctors) were employed to assess the involvement in deciding anesthesia technique from both the patients’ and doctors’ perspectives. Median and interquartile range or standard deviation and mean were used to summarize quantitative variables where appropriate. The analysis of variance test was used to compare the mean scores in different categories. Categorical variables were compared using the chi-square test.

Results

Most patients (93, 60%) preferred a passive role in decision-making regarding the technique of anesthesia. General anesthesia was preferred for both upper and lower limb surgeries. However, the preference for spinal anesthesia increased (56, 47.8% to 95, 81.1%) among those planned for lower limb surgery after the pre-anesthesia evaluation was completed. Patients reported a higher perceived level of shared decision-making (84.44%, 38 out of 45 score) compared to the clinicians’ perspective (71.11%, 32 out of 45 score).

Conclusions

While evaluating shared decision-making regarding anesthesia technique in Indian patients, we found that most patients preferred a passive role, and the perceived level of shared decision-making was found to be higher among patients compared to clinicians.

## Introduction

Shared decision-making (SDM) or patient participation in the decision-making process is one of the three important components of evidence-based medicine. It is a decision-making model with three stages, which consists of bidirectional information exchange, deliberation, and selection of treatment [1]. Through SDM, clinicians can help patients make decisions that are best for them. Previous studies have shown that the majority of patients requiring anesthesia services wish to be involved in the process of decision-making [2]. However, there is limited evidence regarding the level of SDM in anesthesia. The Canadian Anesthesia Research Priority Setting Partnership rated patient involvement in SDM as a high research priority topic [3]. There is a huge unmet need for SDM in India, which has been attributed to a lack of communication, a poor decision-support system, under-resourced healthcare support, and a lack of proper guidelines [4]. Due to a lack of data in the Indian setting, we aimed to evaluate what role (active, collaborative, or passive) patients presenting for elective surgery of a single limb preferred while choosing their anesthesia technique.

## Materials and methods

This prospective, cross-sectional study was conducted at a tertiary care, teaching hospital in South India between August 1, 2018, and August 31, 2019. Patients were consented and recruited after the Institutional Review Board and Ethics Committee clearance was obtained (approval number: 11425). All adults planned for elective surgery of a single upper limb or lower limb, for whom different techniques of anesthesia, such as general anesthesia, spinal, or peripheral nerve block, could be employed, were eligible to participate in the study. Patients with American Society of Anesthesiologists Physical Status > 3, age <18 years, weight >100 kg, known bleeding disorders, neuromuscular weakness, allergic to local anesthetic drugs, chronic alcohol or drug abuse, psychiatric illness, those unable to read or answer the questionnaire, and those undergoing emergency surgery were excluded from the study.

The primary outcome of the study was to determine the level of patient participation (active, collaborative, or passive) while choosing the anesthesia technique [5]. Patient participation in SDM was classified as follows based on the study by Hwang et al. [5]: active role (patients, along with their family, make a decision by themselves), passive role (patients rely on the anesthetist’s decision entirely), and collaborative role (patients prefer to discuss with the anesthetist and make a decision together). The secondary outcomes were to assess patients’ preferences about the anesthesia technique, to compare the decision made regarding the anesthesia technique before and after the pre-anesthesia evaluation, and to compare the difference in the patients’ and doctors’ perception regarding the patient’s involvement in the final decision made.

All study participants filled out a questionnaire, which recorded their demographic details, including age, gender, level of education, previous anesthesia exposure, and source of knowledge of anesthesia. Following this, they underwent the pre-anesthesia evaluation for the proposed surgery. All participants were assessed by the same anesthetist to ensure standardization of care and counseling. The various options for anesthesia were put forward, along with the advantages and disadvantages of each technique. Finally, an informed decision was made regarding the technique of anesthesia, and consent was obtained for the same. The patients’ preferred technique of anesthesia and their preferred role in the process of decision-making regarding the technique of anesthesia were also noted.

After the pre-anesthesia evaluation was completed, all study participants filled out the 9-item Shared Decision-Making Questionnaire (SDM-Q-9) (Appendices) to assess the level of SDM from the patient’s perspective. This standardized questionnaire is a reliable, brief, user-friendly, well-accepted, and commonly used tool to assess patient involvement in medical decision-making. It is self-reported and has demonstrated good internal consistency and construct validity in several studies [6-8]. The questionnaire consists of nine statements, with each statement rated on a six-point Likert scale ranging from completely disagree (scored as 0) to completely agree (scored as 5). The total raw score was calculated by summing the responses to all nine statements, yielding a possible range from 0 to 45. To standardize the analysis, these raw scores were then converted to a 0-100 scale, where a score of 0 represented the lowest level of SDM and 100 indicated the highest possible level [8].

Similarly, the anesthetist in the pre-anesthesia clinic filled the physician version of the SDM-Q-Doc Questionnaire (Appendices) to assess the level of SDM from the clinician’s perspective. It has similar statements to those in the SDM-Q-9 questionnaire, and the analysis of the results followed a similar method [6,9]. A doctor’s score of 28 (25th percentile) was used as a cut-off to define low and high doctors’ SDM scores. Using only the sixth to ninth statements from the questionnaires, a score was calculated separately, as these four statements conveyed more than half of the overall [10]. These scores were also converted to aggregates of 100 to standardize the analysis.

Statistical analysis

The sample size was calculated based on the study by Aggarwal et al., in which 10% of patients played an active role in decision-making [10]. Using the single proportion method, assuming that 10% patients would prefer an active role in decision-making, we would require a sample size of 139 for estimating the expected proportion with 5% absolute precision and 95% confidence. Anticipating a dropout rate of 10%, the sample size was estimated as 153 patients. We could include 156 patients in the given period.

**Figure 1:**
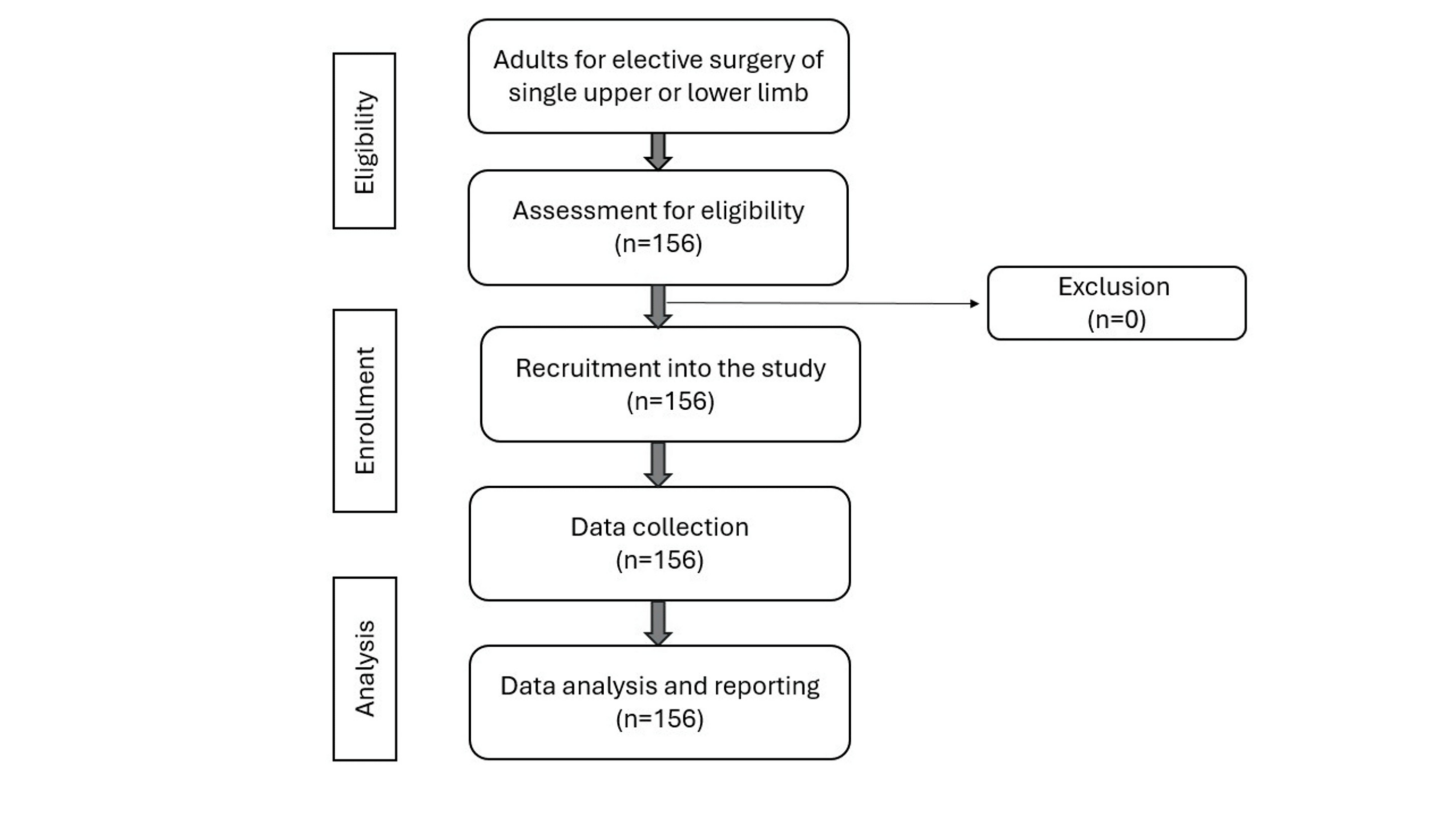
Flowchart depicting the flow of study participants.

Demographic data were reported as frequency distribution and percentages. Quantitative variables were summarized using median and interquartile range or mean and standard deviation, where appropriate. The analysis of variance test was used to compare the mean scores in various categories. The chi-square test was used to compare other clinical and demographic variables if the variables were categorical. The two-sample t-test was used to compare the continuous variables. Correlation between values was calculated using Pearson’s correlation. Data were analyzed using SPSS version 23.0 (IBM Corp., Armonk, NY, USA). Figure 1 illustrates a flowchart of the study methodology.

## Results

The demographic data of the study participants are depicted in Table 1.

**Table 1:** Demographic profile of the study participants.

Parameter	Number	Percentage
Age (in years)		
<30	47	12%
31–40	38	24%
41–50	17	26%
51–60	33	21%
>=60	12	8%
Gender		
Male	117	75%
Female	39	25%
Surgery site		
Upper limb	39	25%
Lower limb	117	75%
Knowledge about anesthesia techniques		
General anesthesia	129	82.70%
Spinal anesthesia	106	67.90%
Peripheral nerve block	40	25.60%
Highest level of education		
Primary school (1^st^–5^th^)	15	9.60%
Middle school (6^th^–10^th^)	40	25.60%
Higher secondary school (11^th^–12^th^)	34	21.80%
College graduate	45	28.80%
Postgraduate/Honors	22	14.10%

The mean age of the participants was 40.49 ± 13.57 years, with 95 (61%) patients having previous anesthesia exposure and 76 (48.7%) citing the previous exposure as their main source of knowledge about anesthesia. The majority (129, 82.7%) of patients knew about general anesthesia and spinal anesthesia (106, 67.9%). However, patients had limited knowledge regarding peripheral nerve blocks (40, 25.6%). The majority of patients (117, 75%) were planned for surgery of the lower limb. The number of patients who had studied beyond school education was 67 (42.9%).

**Figure 2:**
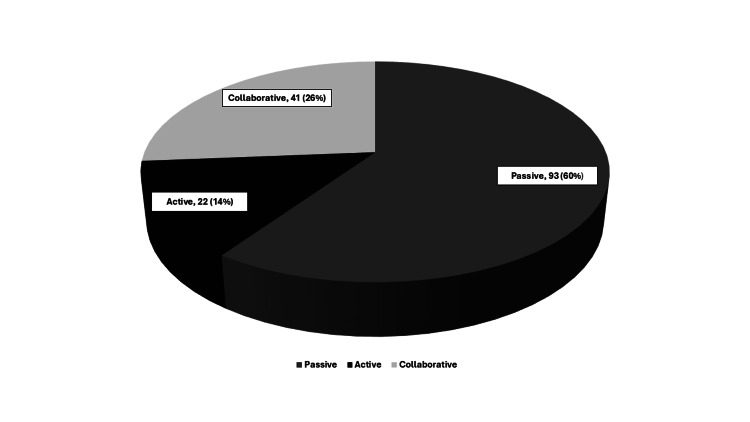
Patients’ preferred role in decision-making.

**Figure 3:**
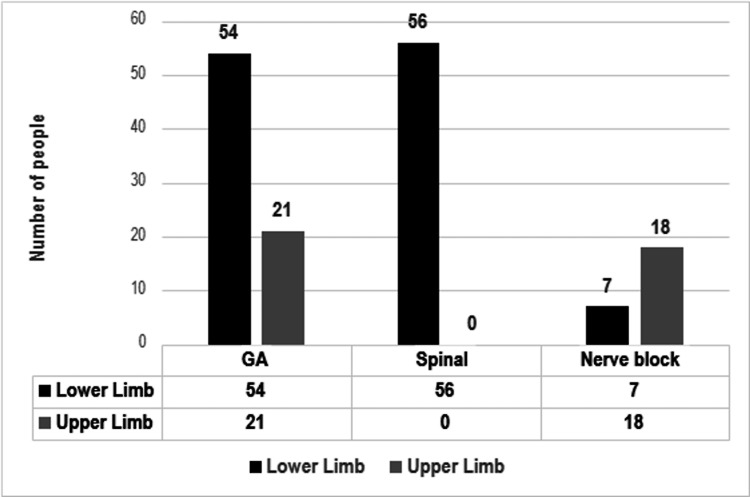
Patients’ preferred anesthesia technique.

Figure 2 depicts the assessment of the preferred role of patients regarding the decision-making process, where the majority of patients chose a passive role, with only 22 (14%) patients choosing an active role. Figure 3 depicts patient preference for general anesthesia, spinal anesthesia, and peripheral nerve blocks for upper limb and lower limb surgeries. Patients’ preferred anesthesia technique before and after their pre-anesthesia evaluation is depicted in Table 2. In our study, the majority of patients planned for lower limb surgery preferred general anesthesia before the pre-anesthesia evaluation, but the preferred choice changed to spinal anesthesia after the pre-anesthesia evaluation (p < 0.001). Among the patients undergoing upper limb surgery, preference for general anesthesia was seen both before and after the pre-anesthesia evaluation (p < 0.05).

**Table 2:** Comparison of the preferred anesthesia technique for lower limb and upper limb surgery before and after the pre-anesthesia evaluation.

Preferred anesthesia technique	Before pre-anesthesia evaluation	After pre-anesthesia evaluation
Lower limb (n = 117)		
General anesthesia	54 (46.1%)	21 (17.9%)
Spinal anesthesia	56 (47.8%)	95 (81.1%)
Peripheral nerve block	7 (5.98%)	1 (0.8%)
Upper limb (n = 39)		
General anesthesia	21 (53.8%)	26 (66.6%)
Peripheral nerve block	18 (46.1%)	13 (33.3%)

Table 3 shows the mean scores of each question from the SDM-Q-9 and SDM-Q-Doc questionnaires. In the group with low doctor scores (≤28), the mean patient score was 30.68, and in the high doctor score group (>28), the patient score was 38.5. This difference was statistically significant (p < 0.001). The analysis of variance test to predict the change in patient scores based on the change in doctors’ scores showed a significant correlation (F-value = 11.517, p < 0.01).

**Table 3:** Mean scores of individual questions from the SDM-Q-9 and SDM-Q-Doc questionnaires. SDM-Q = Shared Decision-Making Questionnaire

	Mean scores from the SDM-Q-9 questionnaire of individual questions			Mean scores from the SDM-Q-Doc questionnaire of individual questions			P-value
	Mean ± SD	Median (maximum total of 5)	95% CI	Mean ± SD	Median (maximum total of 5)	95% CI	
	(maximum total of 5)			(maximum total of 5)			
The doctor made clear that a decision needs to be made	4.09 ± 1.36	5	3.89–4.29	3.70 ± 0.71	4	3.58–3.81	0
The doctor wanted to know exactly how I want to be involved in making the decision	3.78 ± 1.61	4	3.53–4.01	3.25 ± 0.99	3	3.08–3.40	0
The doctor said that there are different options for treating my medical condition	3.83 ± 1.62	5	3.56–4.09	3.44 ± 1.04	4	3.27–3.60	0
The doctor precisely explained the advantages and disadvantages of the treatment options	3.31 ± 0.15	4	3.0–3.62	2.26 ± 1.38	2	2.03–2.48	0
The doctor helped the patient understand all the information	4.38 ± 0.85	5	4.24–4.51	3.87 ± 0.48	4	3.79–3.94	0
The doctor asked the patient which treatment option they preferred	4.26 ± 1.17	5	4.06–4.44	3.86 ± 0.62	4	3.76–3.96	0
The doctor and patient thoroughly weighed the different treatment options	4.04 ± 1.27	4.5	3.81–4.23	3.15 ± 1.09	3	2.99–3.32	0
The doctor and patient selected a treatment option together	4.16 ± 1.13	5	3.99–4.33	3.74 ± 0.70	4	3.63–3.86	0
The doctor and patient reached an agreement on how to proceed	4.26 ± 1.02	5	4.10–4.41	4.01 ± 0.47	4	3.94–4.08	0

While 127 (79.2%) participants reported being informed by their doctor about the available anesthesia options, only 107 (68.5%) stated that they were informed of the advantages and disadvantages of each technique. In contrast, according to the doctors, anesthesia options were explained to 125 (83.2%) patients, but the benefits and risks were communicated to only 71 (45.55%) patients.

## Discussion

Patient participation is an important component of evidence-based medicine. SDM integrates clinical expertise, current research evidence, and patient preferences to provide the most appropriate treatment for each. However, multiple studies have shown that patient involvement is often suboptimal and does not fully meet its intended purpose [4,11]. Although there is a massive scope for the application of the principles of SDM in the field of anesthesiology, this concept has not been explored in the Indian context. This study was designed to address this unmet need in the anesthesiology literature.

Patient involvement in medical decision-making depicts geographical variations. Large population-based studies from Europe report that about half of the patients were involved in the decision-making process [12]. Reports from the Asian population showed that more than half of the patients would prefer shared decision-making (before consultation), but fewer patients felt that a shared decision was made during the consultation, revealing a discrepancy between patients’ expectations and the doctors’ perception of patients’ preferred role [12].

Our results suggest that the majority of the patients preferred a passive role rather than a collaborative or active role in deciding the anesthesia technique. A preference for a passive role in decision-making in our setting could be attributed to a lack of awareness of the possible alternative treatment options (peripheral nerve blocks) and patients having a high degree of trust in their doctors [13].

While looking at patient involvement in decision-making in rural India, patients were seen to rely on the doctor to make the decision for them [14]. A study among Asian patients with cancer reported that in all the countries studied, no patient involvement was the most commonly reported, perceived, and preferred decision-making role [15]. The study found that patients were not interested in SDM, and socially disadvantaged groups, such as females, lower educated, and those from minority groups, were more likely to report lower levels of SDM [15]. In contrast, among well-educated urban Caucasian women with ovarian cancer, the majority wished to share the medical decision-making with their doctor [16]. However, patient involvement in decision-making has been shown to improve satisfaction with anesthesia services [17].

**Figure 4:**
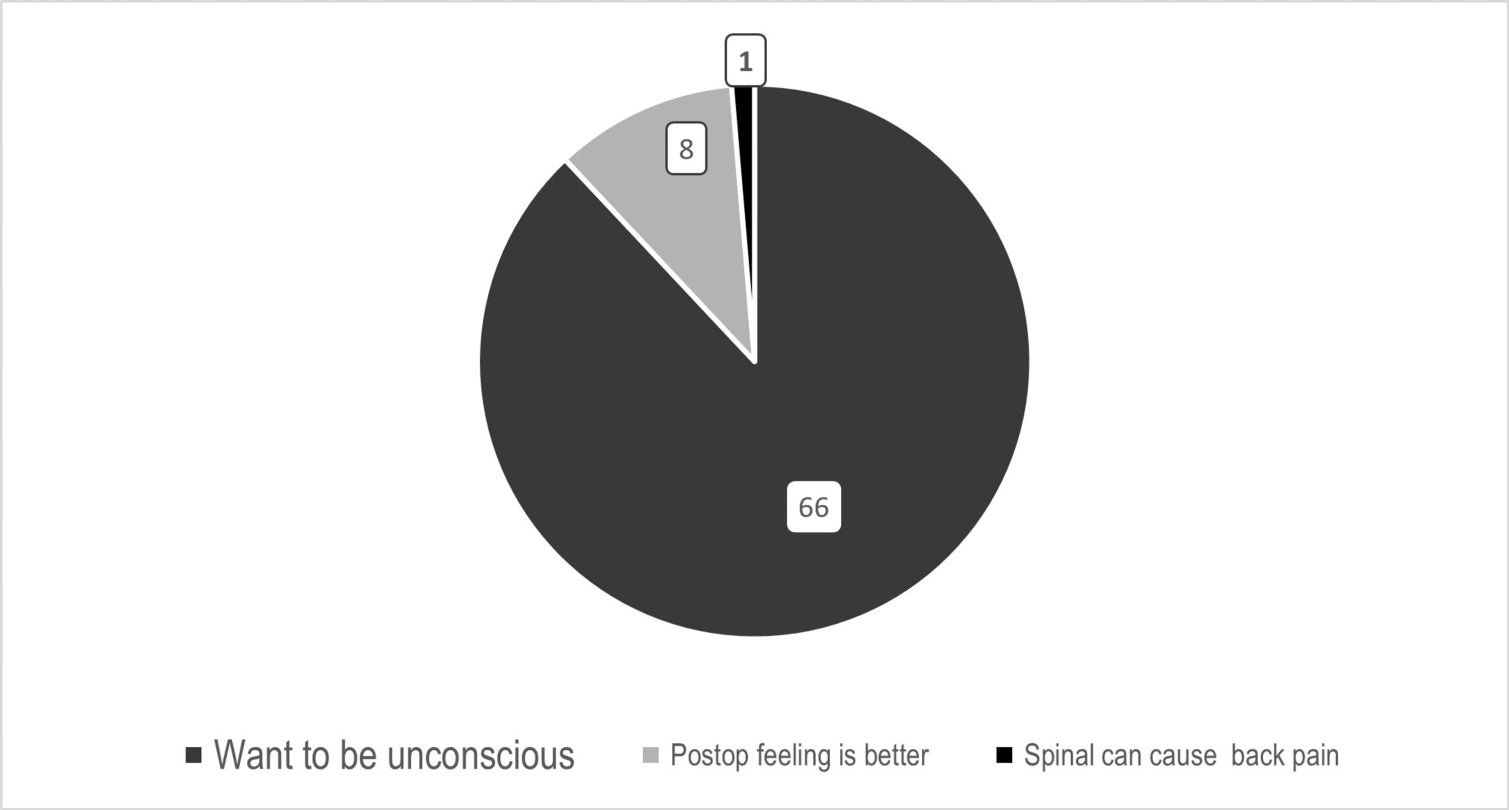
Reason for the preference of general anesthesia (n=75).

While looking at patient preference for an anesthesia technique, our findings showed that, irrespective of the site of surgery, there was a preference for general anesthesia, which was in concordance with the results of other similar studies [18,19]. Most patients opting for general anesthesia did not want to be conscious during the surgery (66, 80%) (Figure 4). Reasons cited for preference of general anesthesia over regional anesthesia in other studies were pain at the puncture site [5] and fear of needles or of being awake during the surgery [19,20].

Moreover, after completion of the pre-anesthesia evaluation, we found that more patients preferred spinal anesthesia among those undergoing lower limb surgery. These findings suggest that the discussion of the risks and benefits of each anesthesia technique during the pre-anesthesia visit can influence the patients’ decision regarding the choice of anesthesia. Flierler et al. concluded that more emphasis should be placed on patient involvement in decision-making during the pre-anesthesia visits [2]. A study among chronic pain patients concluded that the desire for information was high, with higher levels of SDM in pre-medication visits compared to the chronic pain clinic, especially among those with higher levels of education [21]. As the pre-anesthesia encounter offers an ideal opportunity to guide patients’ choice of anesthesia technique, our study led to the introduction of a patient information sheet to provide more information regarding regional anesthesia in our setting.

Compared with our results (Table 4), Stubenrouch et al. assessed the level of SDM using the SDM-Q-9 and the SDM-Q-Doc questionnaires and reported higher scores in patients (median scores of 91.7% vs. 84.44% in our study) and the clinician category (84.3% vs. 71.11% in our study). [11] This discrepancy in scores by Stubenrouch et al. and our study can be explained by a higher exposure to previous anesthesia in their patient cohort (68, 85% vs. 95, 61% in our study). Additionally, at the time of the conduct of this study, we provided only verbal information regarding the anesthesia technique, as patient information sheets were unavailable in our department.

**Table 4:** Comparison of total level of shared decision-making as perceived by the patient and the doctor from SDM-Q-9 and SDM-q-Doc questionnaires. SDM-Q = Shared Decision-Making Questionnaire

	Patient score		Doctor score		P-value
	Mean ± SD (% score)	Median	Mean ± SD (% score)	Median	
Questions 1–9 (total score = 45)	36.12 ± 9.28 (80.26%)	38.0 (84.44%)	31.28 ± 4.77 (69.52%)	32.0 (71.11%)	<0.001
Questions 6–9 (total score = 20)	16.7 ± 4.04 (83.6%)	18.0 (90%)	14.8 ± 1.96 (73.9%)	15.0 (75%)	<0.001

Regarding the perceived level of SDM, we found that SDM was higher among patients than among doctors. Similarly, Williams et al. reported that although patients and doctors were positively inclined toward the concept of SDM, the actual engagement in SDM was still lagging behind the attitude toward the process [22].

The strength of our study was that a single doctor was involved in the pre-anesthesia evaluation and data collection for all the patients, thereby eliminating chances of interobserver variability and bias. However, this may not fully represent all doctors from this center and cannot be generalized. Other limitations could be that the questionnaires used were subjective and could have been influenced by various factors, such as the time taken for the evaluation in the pre-anesthesia clinic, the doctors’ tone, patients’ previous experiences, and their understanding of the study and its objectives. Additionally, we cannot rule out recall bias regarding the anesthesia technique used during previous exposure.

While most of our patients preferred a passive role in the decision-making process about their anesthesia technique for elective surgery on a single limb, the pre-anesthesia encounter did result in a change in patients’ preferences in some instances. The results from our study indicate a need to explore the reasons for the preference for a passive decision-making role by the patients. Further studies are required to elucidate factors that may affect the patients’ decision regarding anesthesia technique. Future studies can also explore the impact of patient information material on their preference for the anesthesia technique.

## Conclusions

Our results indicate a significant gap between the perceived level of SDM between patients and anesthesiologists. We suggest the adoption of decision aids in selecting anesthesia techniques to help fulfill the unmet needs that have been highlighted in our study.

## Figures and Tables

**Figure 5:**
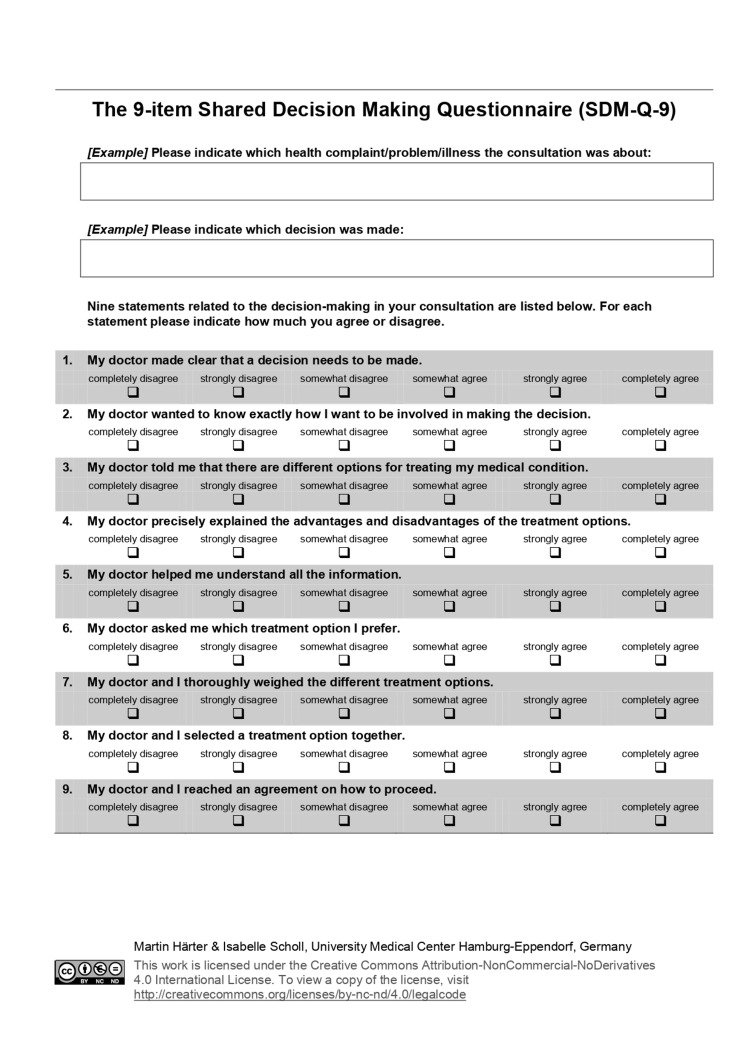
SDM-Q-9 questionnaire.

**Figure 6:**
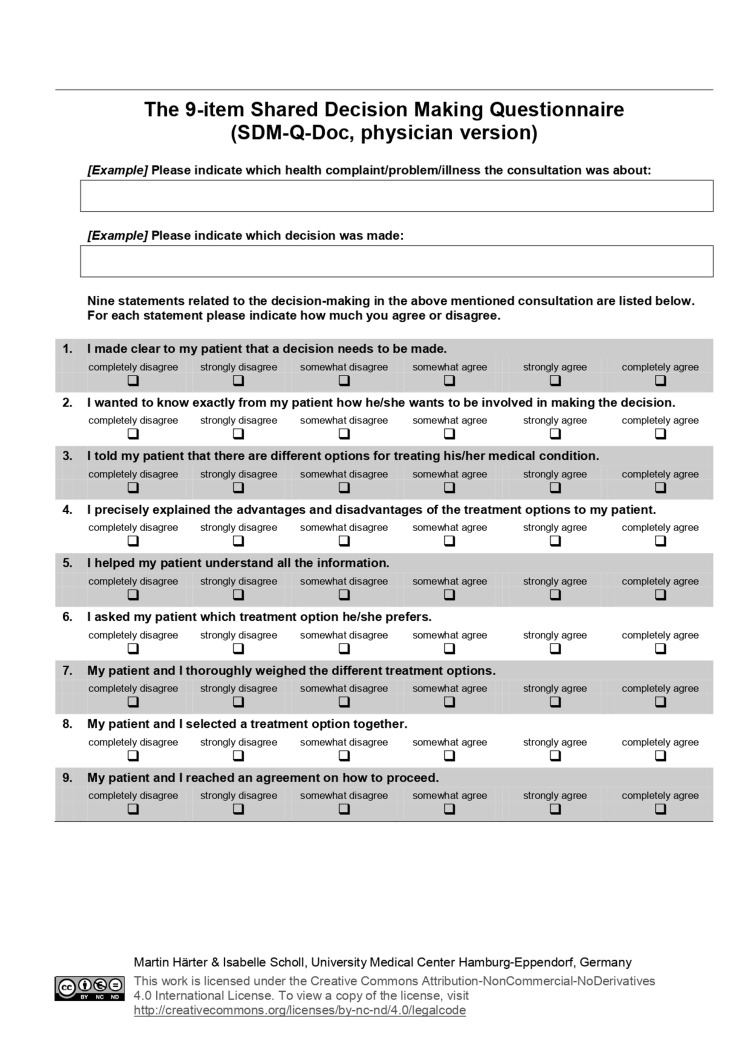
SDM-Q-Doc questionnaire.
